# Advancements and trends in exosome research in lung cancer from a bibliometric analysis (2004-2023)

**DOI:** 10.3389/fonc.2024.1358101

**Published:** 2024-04-16

**Authors:** Wen Zhong, Xiaofei Zhao, Xiabiao Zhang, Yiwen Xu, Mengqian Liu, Xiaoyun Yang, Yi Jiang, Xiaozhu Shen

**Affiliations:** ^1^Department of Geriatrics, Lianyungang Hospital Affiliated to Jiangsu University, Lianyungang, China; ^2^Department of Neurosurgery, Jiangxi Provincial People’s Hospital, The First Affiliated Hospital of Nanchang Medical College, Nanchang, China; ^3^Department of Infectious Disease, The First Affiliated Hospital of Ningbo University, Ningbo, China; ^4^Department of Geriatrics, Lianyungang Hospital Affiliated to Bengbu Medical College, Lianyungang, China

**Keywords:** lung cancer, exosomes, bibliometrics, knowledge map, CiteSpace

## Abstract

**Background:**

Lung cancer, characterized by its high morbidity and lethality, necessitates thorough research to enhance our understanding of its pathogenesis and discover novel therapeutic approaches. Recent studies increasingly demonstrate that lung cancer cells can modulate the tumor microenvironment, promoting tumor growth, and metastasis through the release of exosomes. Exosomes are small vesicles secreted by cells and contain a variety of bioactive molecules such as proteins, nucleic acids, and metabolites. This paper presents a comprehensive review of exosome research in lung cancer and its progress through bibliometric analysis.

**Methods:**

Publications related to exosomes in lung cancer patients were systematically searched on the Web of Science Core Collection (WoSCC) database. Bibliometric analysis was performed using VOSviwers, CiteSpace, and the R package “Bibliometrics”. Publications were quantitatively analyzed using Microsoft Office Excel 2019. The language of publication was restricted to “English” and the search strategy employed TS=(exosomes or exosomes or exosomes) and TS=(lung cancer). The search period commenced on January 1, 2004, and concluded on November 12, 2023, at noon. The selected literature types included Articles and Reviews.

**Results:**

The study encompassed 1699 papers from 521 journals across 71 countries and 2105 institutions. Analysis revealed a consistent upward trend in lung cancer exosome research over the years, with a notable surge in recent times. This surge indicates a growing interest and depth of inquiry into lung cancer exosomes. Major research institutions in China and the United States, including Nanjing Medical University, Shanghai Jiao Tong University, Chinese Academy Of Sciences, and Utmd Anderson Cancer Center, emerged as crucial research hubs. The annual publication count in this field witnessed a continuous rise, particularly in recent years. Key terms such as lung cancer, non-small cell lung cancer (NSCLC), microvesicles, intercellular communication, exosomal miRNAs, and oncology dominated the research landscape. Fields like cell biology, biochemistry, biotechnology, and oncology exhibited close relation with this research. Clotilde Théry emerged as the most cited author in the field, underlining her significant contributions. These results demonstrate the broad impact of exosome research in lung cancer, with key terms covering not only disease-specific aspects such as lung cancer and NSCLC but also basic biological concepts like microvesicles and intercellular communication. Explorations into exosomal microRNAs and oncology have opened new avenues for lung cancer exosome research. In summary, lung cancer exosome research is poised to continue receiving attention, potentially leading to breakthroughs in treatment and prevention.

**Conclusion:**

Publications on lung cancer exosomes show a rising trend year by year, with China and the United States ranking first and second in terms of the number of publications. However, there is insufficient academic learning cooperation and exchanges between the two sides, and Chinese universities account for a large proportion of research institutions in this field. Jing Li is the most productive author, Clotilde Théry is the most co-cited author, and Cancers is the journal with the highest number of publications. The current focus in the field of lung cancer exosomes is on biomarkers, liquid biopsies, immunotherapy, and tumor microenvironment.

## 1 Introduction

Lung cancer is a major global health challenge, and as one of the most lethal cancers, its impact is widespread and far-reaching (1). This is particularly true of non-small cell lung cancer (NSCLC), which not only accounts for the vast majority of lung cancer cases, over 85%, but also has an alarming mortality rate of 80% to 90% (2). According to the Annual Cancer Report 2023, lung cancer ranks as the leading cause of death from all cancers, with an average of approximately 350 deaths per day, a figure that is almost two and a half times higher than the second deadliest type of cancer—colorectal cancer (3). This figure highlights the serious public health threat posed by lung cancer. Despite the progress made in recent years in diagnostic techniques, treatment methods, and prognostic assessment of lung cancer, the incidence and mortality rates of lung cancer remain high. Therefore, the search for new biomarkers and new targets for targeted therapy is crucial for the early diagnosis and clinical treatment of lung cancer (4).In recent years, more and more studies have found that exosome vesicles (EVs) play an important role in the occurrence, development, and treatment of lung cancer (5–7). A large number of studies have found that EVs can influence the occurrence and development of lung cancer by interacting with the tumor microenvironment, mediating the growth and metastasis of tumor cells, tumor immunomodulation, and resistance to radiotherapy (8, 9). In addition, EVs can provide important information for the early diagnosis of lung cancer and may become a tumor marker for lung cancer (10). Therefore, studying EVs in lung cancer will provide new ideas for the diagnosis and treatment of lung cancer. This paper aims to explore the developmental dynamics, research hotspots, and trends of EVs in lung cancer research through bibliometric analysis, to a comprehensive summarize and prospect the development direction of this field, to provide relevant researchers and clinicians with comprehensive understanding and ideas, and to promote the in-depth development of EVs in lung cancer research.

## 2 Methods

### 2.1 Research purpose

To analyze the dynamics and evolution trajectory of lung cancer-related exosomes over the past two decades, this paper examines journal literature, primarily sourced from the scientific research cooperation network and literature databases. Employing keyword-level information integration and data mining, the research aims to summarize the developmental characteristics of lung cancer-related exosome studies, offering an in-depth understanding of the field’s dynamics and focus. This insight is of considerable significance for researchers engaged in relevant theoretical research.

### 2.2 Data sources

**Figure 1:**
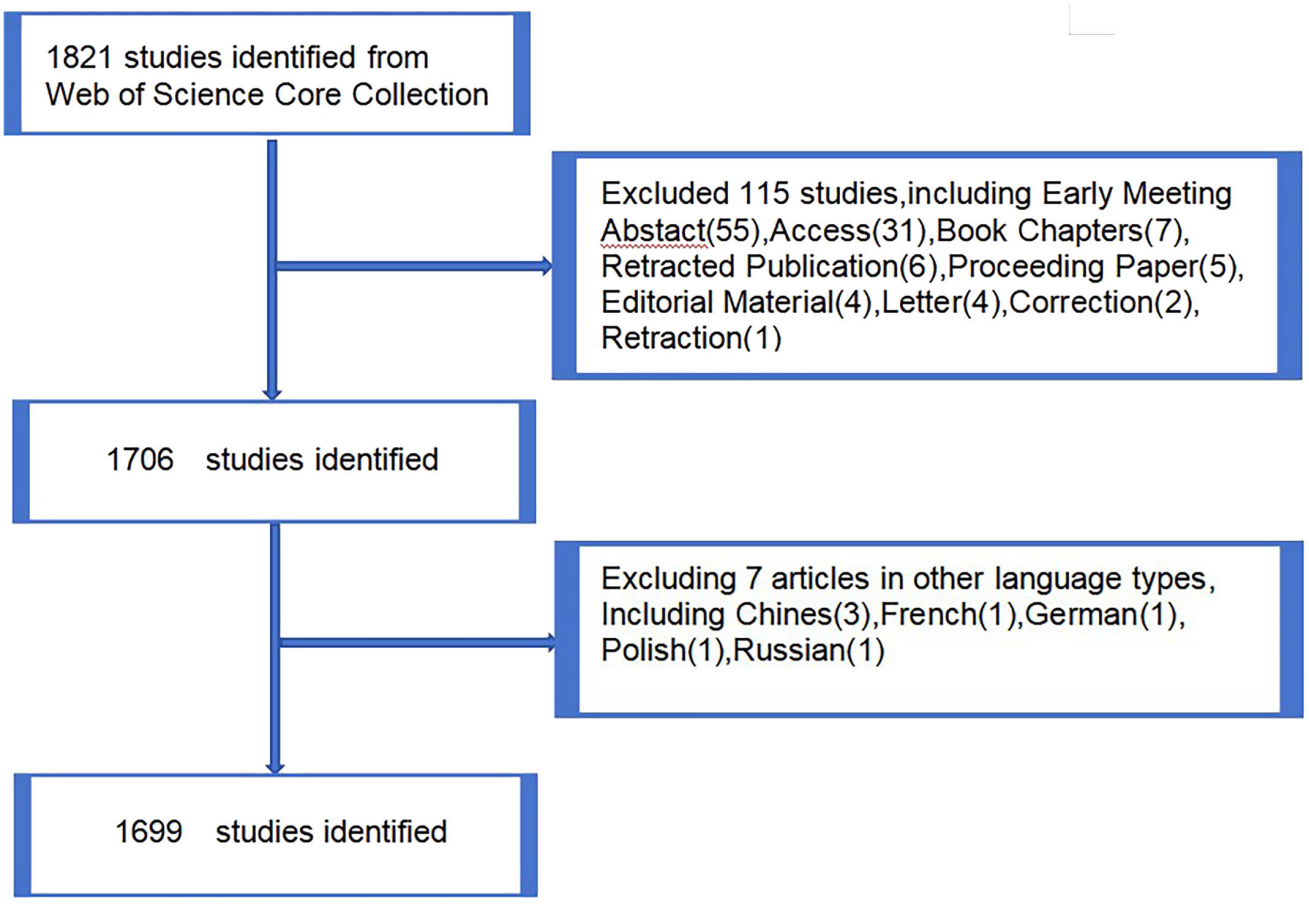
Publications screening flowchart.

The Web Science Core (https://www.webofscience.com/wos/woscc/basicsearch) collection was selected as the data source for its excellent digital literature resources and is widely recognized as the most suitable database for bibliometric studies. A search of exosome-related literature on lung cancer from 2004 to 2023 was conducted on the Web of Science database, with the search deadline set at noon on November 12, 2023. The search strategy used was ts=(exosome or exosome or exosome), ts=(lung cancer), and LA=(English), yielding a total of 1,821 documents. During the screening process, Article and Review as the literature types, excluding other types such as Early Meeting Abstract (55), Access (31), Book Chapters (7), Retracted Publication (6), Proceeding Paper (5), Editorial Material (4), Letter (4), Correction (2), and Retractions (1). Finally, 1699 valid papers were included (Article (1217), Review (482). Data including title, year of publication, journal, author, institution, country, study name, citation, and study type were collected. Two researchers ensured data accuracy and study reproducibility by downloading and analyzing the data using Microsoft Excel and Endnote software (**Figure 1**).

### 2.3 Research methods and tools

Initially, 1699 journal papers were screened based on the number of journal publications and impact factors in the lung cancer exosomes field. Subsequently, the data were analyzed and visualized using VOSviewer, CiteSpace, and R. VOSviewer is a software tool for visualizing and analyzing bibliometric networks. It is commonly used to explore and understand the structure of the scientific literature and identify key authors, institutions, and topics in a specific research area (11). VOSviewer allows the importation of bibliographic data from sources such as Web of Science, Scopus, and PubMed, generating visualizations of co-author networks, co-citation networks, and keyword co-occurrence networks. These maps can be customized and manipulated to highlight different aspects of the data (12). In addition to visualizing the network structure, VOSviewer offers a variety of analytical tools to help users gain insights from their data. These tools include cluster analysis (grouping similar items) and density visualization (showing the strength of connections between items in a network) (13). CiteSpace is another software tool developed by Chao-Mei Chen of Drexel University, which was used for visualizing and analyzing scientific literature in bibliometrics and scientometrics (14). It is widely used in academic research to identify trends, patterns, and relationships in large collections of research papers. CiteSpace uses network analysis and visualization techniques to map the knowledge structure of a research field, identify key concepts and themes, and trace the evolution of research over time. It is also capable of generating bibliometric metrics, such as citation counts and co-citation networks, to assess the impact and influence of individual papers, authors, and journals (15). For thematic evolution analysis, the R package “bibliometrix” (version 4.2.3) (https://www.bibliometrix.org) was employed (16). Additionally, a global distribution network map of exosome publications related to lung cancer was constructed. Quantitative analysis of the publications was performed using Microsoft Office Excel 2019.

## 3 Research results and analysis

### 3.1 Distribution of papers and analysis of published journals

**Figure 2:**
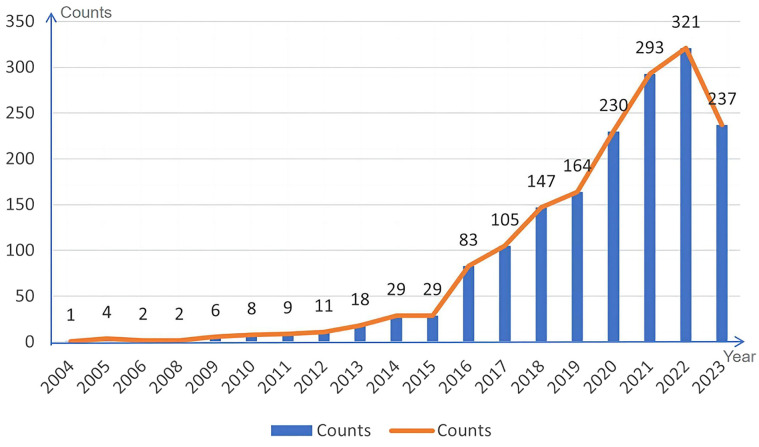
Annual output distribution of exosome papers related to lung cancer.

A descriptive analysis of the annual output of the 1699 journal articles on exosome research related to lung cancer after data cleaning is depicted in **Figure 2**.

As illustrated in **Figure 2**, there were fewer outcomes related to exosome research associated with lung cancer from 2004 to 2008. This can be attributed to two primary factors. Firstly, the global research and cognition of exosomes were relatively limited during this time, with many techniques and methods still in their developmental stages. Secondly, there were limitations related to technical tools, such as experimental equipment, software, and data availability. Between 2009 to 2014, there was a gradual increase in the volume of literature on exosome research related to lung cancer, indicating a growing interest in the field. However, the number of publications remained relatively small, with an average of approximately 13 papers per year. This suggests that research on exosomes in lung cancer was still in its infancy during this period. From 2015 -2022, the literature in this field exhibited a sharp upward trend. The results of exosome research in lung cancer experienced a significant increase in both quantity and influence. By 2022, the number of publications has surged to 321 papers, reaching a new height. Notable international influential journals contributing significantly to lung cancer exosome research include Nature, Cancer Research, Journal of EVs, and so on.

### 3.2 Statistics on the distribution of high-yield countries for lung cancer exosome research

**Figure 3:**
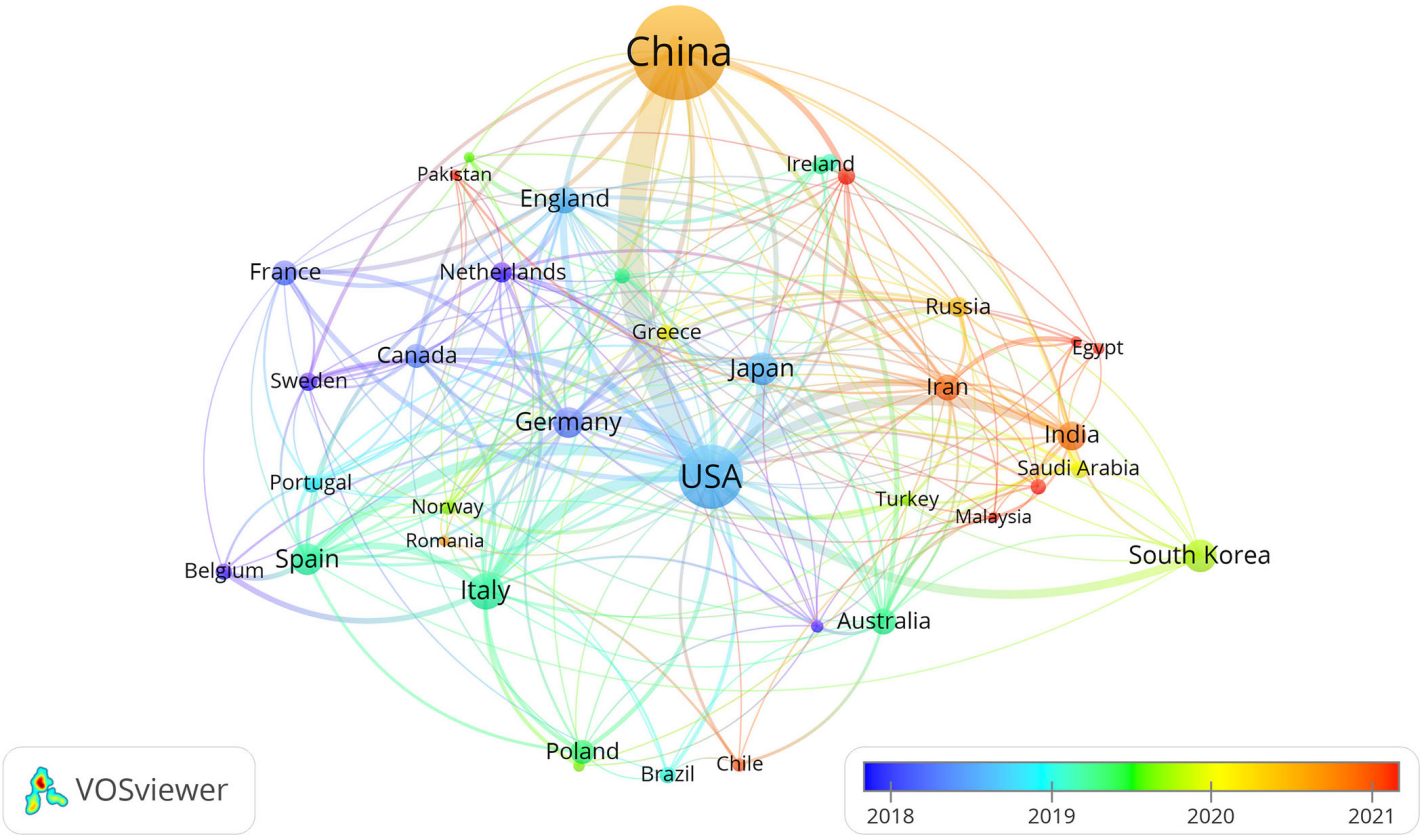
Visual knowledge map of high-yield countries for lung cancer exosome research.

By selecting the “Country” option in the settings panel of the VOSviewer software and maintaining the system defaults, a knowledge graph of the world’s high-yield lung cancer exosome research countries is generated, as detailed in **Figure 3**. Each node in the graph represents a country, with connecting lines representing links between two countries, and the node size corresponds to the number of issued papers. For a better understanding of the node hierarchy in the field for deeper data mining, see **Table 1** for detailed insights.

**Table 1:** Top 10 high-yield countries ranked by the number and centrality of the paper.

Rank	Country	Documents	Country	Centrality
1	China	892 (52.5%)	USA	0.44
2	USA	345 (20.3%)	Iran	0.21
3	Italy	90 (5.3%)	Australia	0.16
4	Japan	69 (4.1%)	Italy	0.13
5	South Korea	69 (4.1%)	Germany	0.11
6	Spain	64 (3.8%)	England	0.10
7	Germany	8 (3.4%)	Saudi Arabia	0.10
8	India	48 (2.8%)	China	0.08
9	England	41 (2.4%)	India	0.07
10	Iran	40 (2.4%)	Qatar	0.07

**Figure 4:**
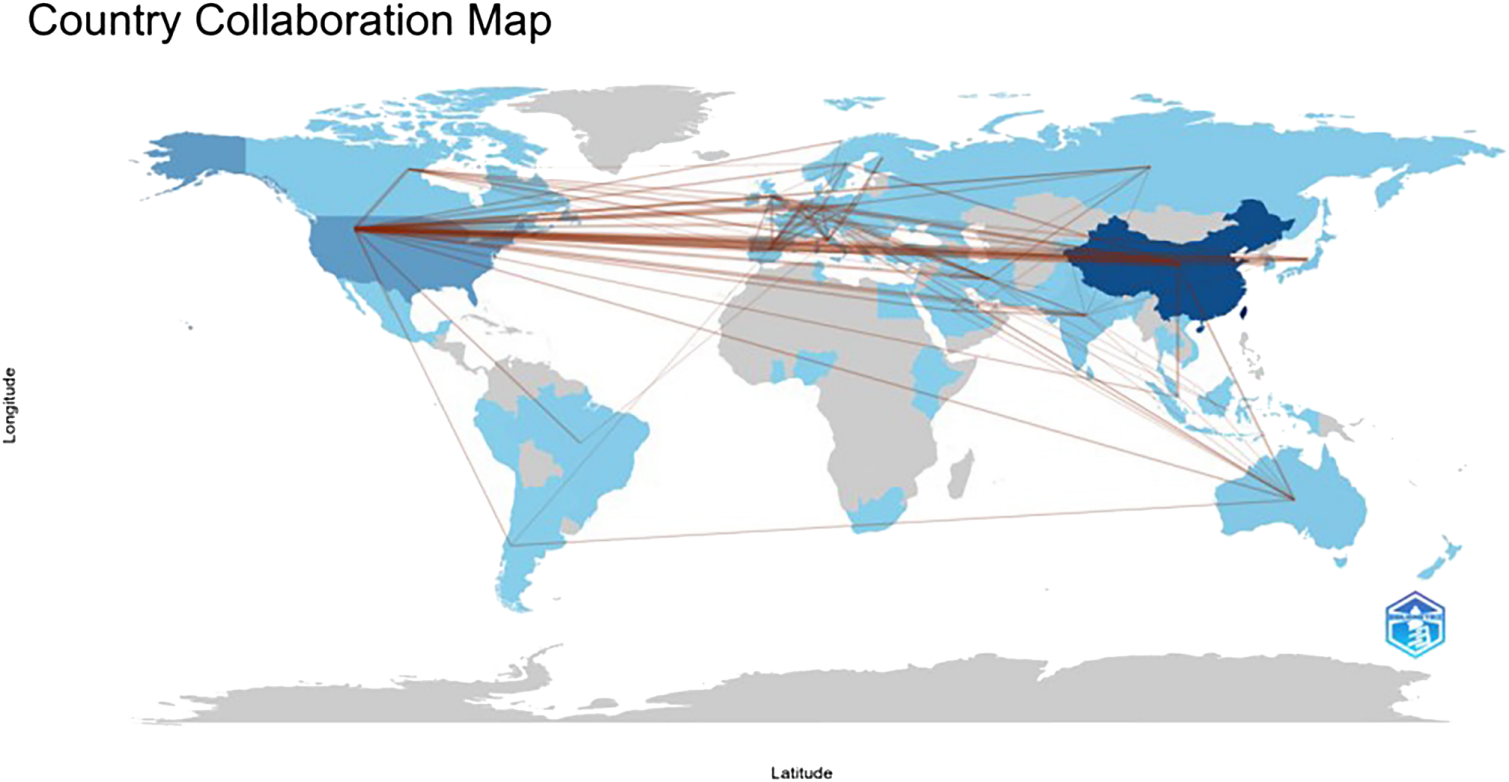
The geographical distribution of high-yield countries for lung cancer exosome research.

In terms of paper output, China, the United States, and Italy emerge as the leading contributors to articles in the field of lung cancer exosomes, followed by seven countries with relatively smaller differences in paper output. **Figure 3** illustrates that China initiated research on lung cancer exosomes comparatively late. However, when compared with France, Sydney, Canada, New Zealand, Germany, the United Kingdom, the United States, Japan, and other countries, there is a noticeable advancement and depth in its research. Examining the centrality degree, nodes with centrality values greater than or equal to 0.1 are considered critical nodes, signifying important factors influencing changes in the research field. As shown in **Table 1**, seven countries, namely the United States, Ireland, Australia, Italy, Germany, the United Kingdom, and Saudi Arabia, demonstrate centrality values greater than or equal to 0.1, portraying high innovation and impactful roles in the field of lung cancer exosomes. Using a criterion of publications greater than or equal to 2, we filtered and visualized 37 countries, creating a collaborative network (see **Figure 4**). In this network, each node represents a country, and connections between nodes indicate shared publications of two or more. Notably, positive partnerships are observed in this collaborative network. China exhibits close collaborative relationships with countries such as the United States, Germany, Japan, and Singapore. Similarly, the United States demonstrates positive collaborations with Italy, Japan, India, and Spain. This visualization of collaborative networks provides a clear depiction of the scientific cooperation landscape among different countries. This visual presentation facilitates an understanding of the dynamics of global scientific collaborations targeting exosome research in lung cancer, providing a collaborative foundation for future research.

### 3.3 Distribution statistics of high-yield institutions for lung cancer exosome research

**Figure 5:**
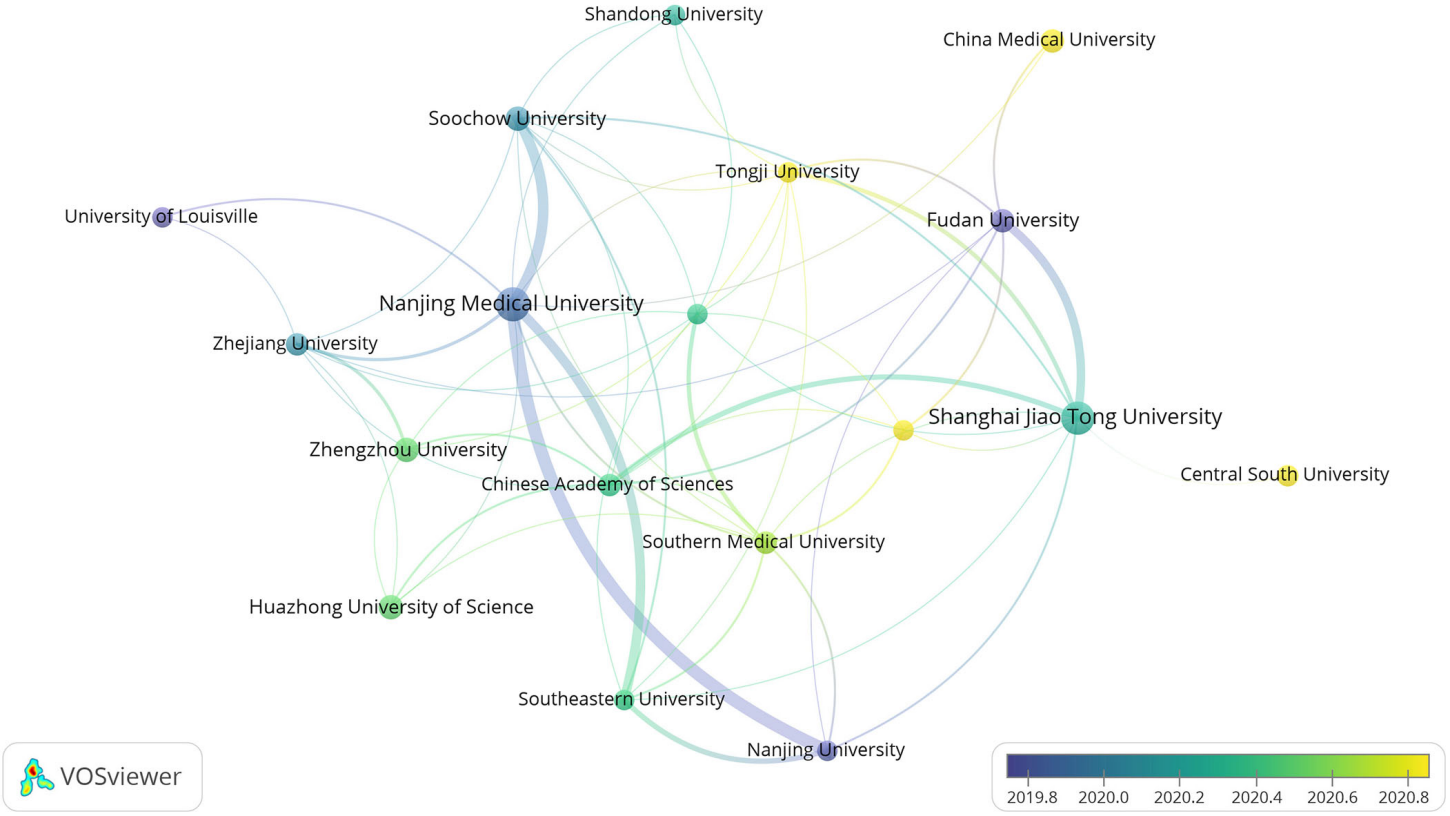
Cooperation map of high-yield institutions.

Running the VOSviewer software and selecting the “Institution” option generates a map illustrating the distribution of high-yield organizations (**Figure 5**). The larger circles in the map do not necessarily correspond to more connecting lines, indicating that organizations with more research results are not necessarily closely related to other organizations.

The high-yield institutional collaboration graph comprises 2104 nodes and 2095 connecting lines. Each node represents the number of papers produced by the institution while connecting lines indicate mutual collaboration among organizations. A higher degree of collaboration is reflected by more connecting lines, signifying a greater level of cooperation between the respective organizations. **Figure 5** reveals a closer collaboration among organizations enabling the comprehensive utilization of literature from various universities and research institutions. This collaborative approach stimulates the exploration of new research horizons, facilitating more profound and expedited meaningful research. Primarily, the issuing institutions are colleges and universities or research institutes, with high-yield research institutes including Nanjing Medical University, Shanghai Jiao Tong University, Chinese Academy Of Sciences, Utmd Anderson Cancer Center, and so on. Notably, Chinese institutions account for 60% of the top ten in terms of frequency. However, Chinese universities and research institutes do not exhibit extensive collaboration with other institutions, indicating a predominant reliance on internal resources for independent research.

### 3.4 Statistics on the distribution of highly productive authors and co-cited authors of lung cancer exosome

**Figure 6:**
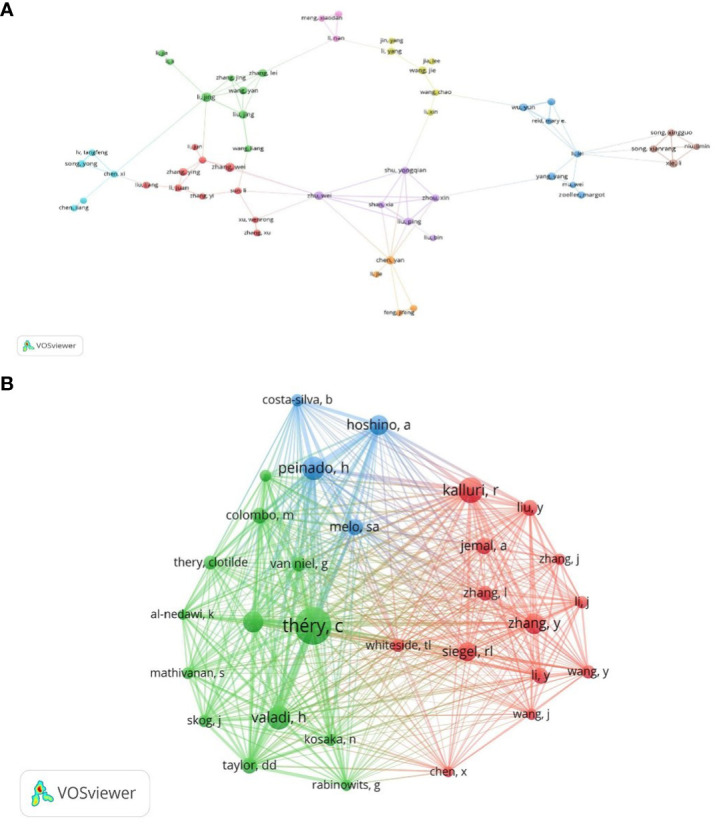
The visualization of authors **(A)** and co-cited authors **(B)** on research of exosomes in lung cancer.

To better reflect the core authorship and relevance in the field of lung cancer exosomes, author collaboration mapping was visualized for 1,699 papers (**Figure 6A**).

In **Figure 6A**, node sizes represent the number of papers published by authors, and connecting lines symbolize mutual collaborative relationships. According to Price’s law, assuming the number of papers by the most productive authors in a given field is nmax, then m=0.749nmax1/2. In this field, authors who have published more than m article are considered the primary authors. In our study, nmax=13, and m≈3, thus, authors who have published more than 3 articles are considered the core authors. We identified 105 core authors in the sample literature, with top-ranked authors including Jing Li, Takahiro Ochiya, Wei Wang, and Wei Zhang. Among 17137 co-cited authors, 4 authors have been co-cited more than 300 times (**Table 2**). The most co-cited author was Clotilde Théry (n=502), followed by Raghu Kalluri (n=338), Hadi Valadi (n=318), and Héctor Peinado (n=310). Authors with a minimum co-citation count of 50 were filtered to map the co-citation network (**Figure 6B**). As shown in **Figure 6B**, there are active collaborations between different co-citing authors, such as Clotilde Théry, Raghu Kalluri, Héctor Peinado, and Rebecca L Siegel. Overall, collaborations in the field of exosomes in lung cancer are robust, with research power concentrated, and close ties among individual scholars are important for in-depth exploration of the field.

**Table 2:** Top 10 authors and co-cited authors on research of exosomes in lung cancer.

Rank	author	documents	Co-Cited Authors	citations
1	Jing Li	12	Clotilde Théry	502
2	Takahiro Ochiya	12	Raghu Kalluri	338
3	Wei Wang	11	Hadi Valadi	318
4	Wei Zhang	11	Héctor Peinado	310
5	Farrukh Aqil	10	Graça Raposo	278
6	Wei Zhu	10	Yan Zhang	275
7	Yan Chen	9	Ayuko Hoshino	268
8	Jing Liu	9	Rebecca L Siegel	255
9	Radha Munagala	9	Melo Sa	221
10	Sunitha Nagrath	9	Ahmedin Jemal	217

### 3.5 Periodicals and syndicated journals

**Figure 7:**
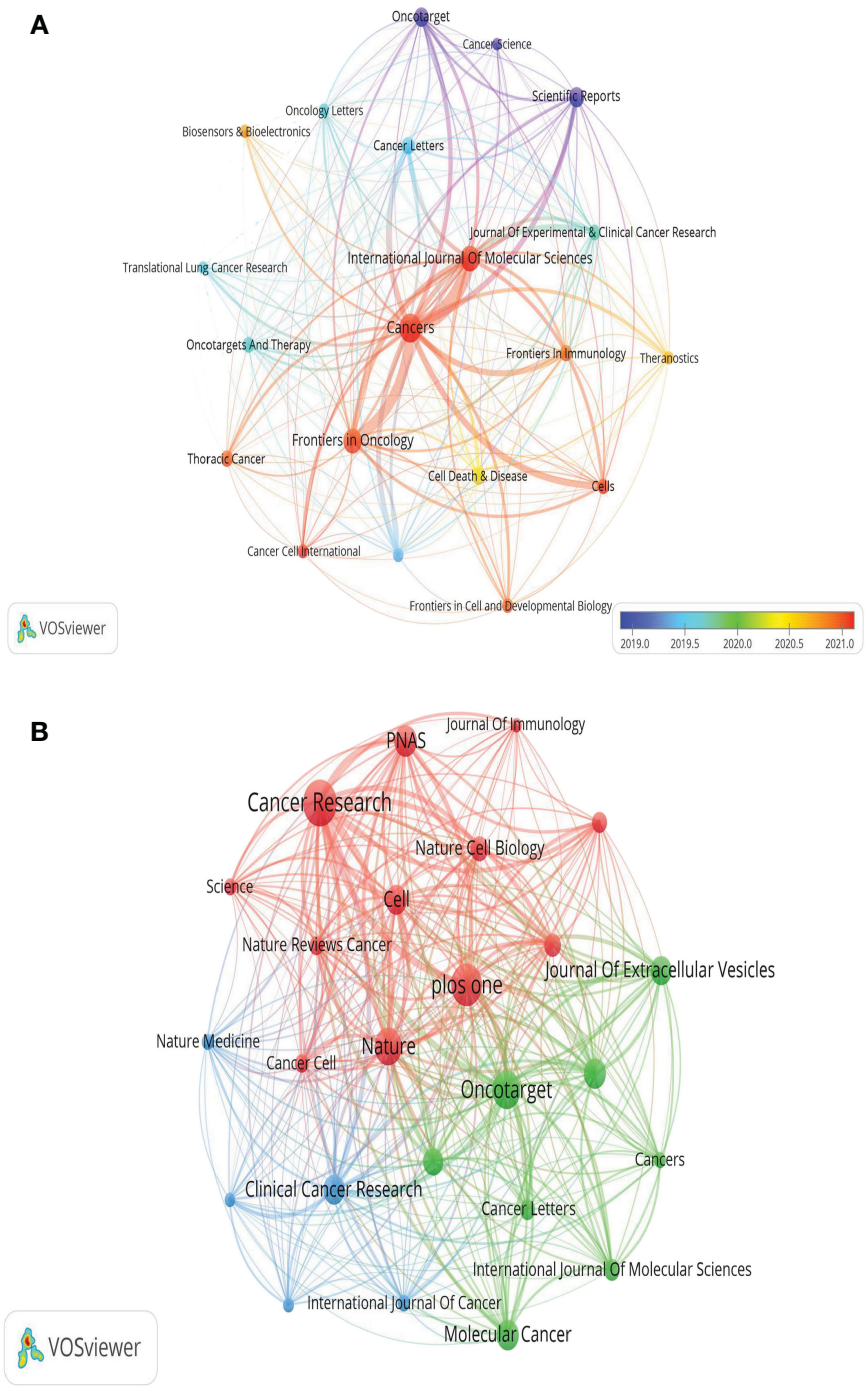
The visualization of journals **(A)** and co-cited journals **(B)** research of exosomes in lung cancer.

**Figure 8:**
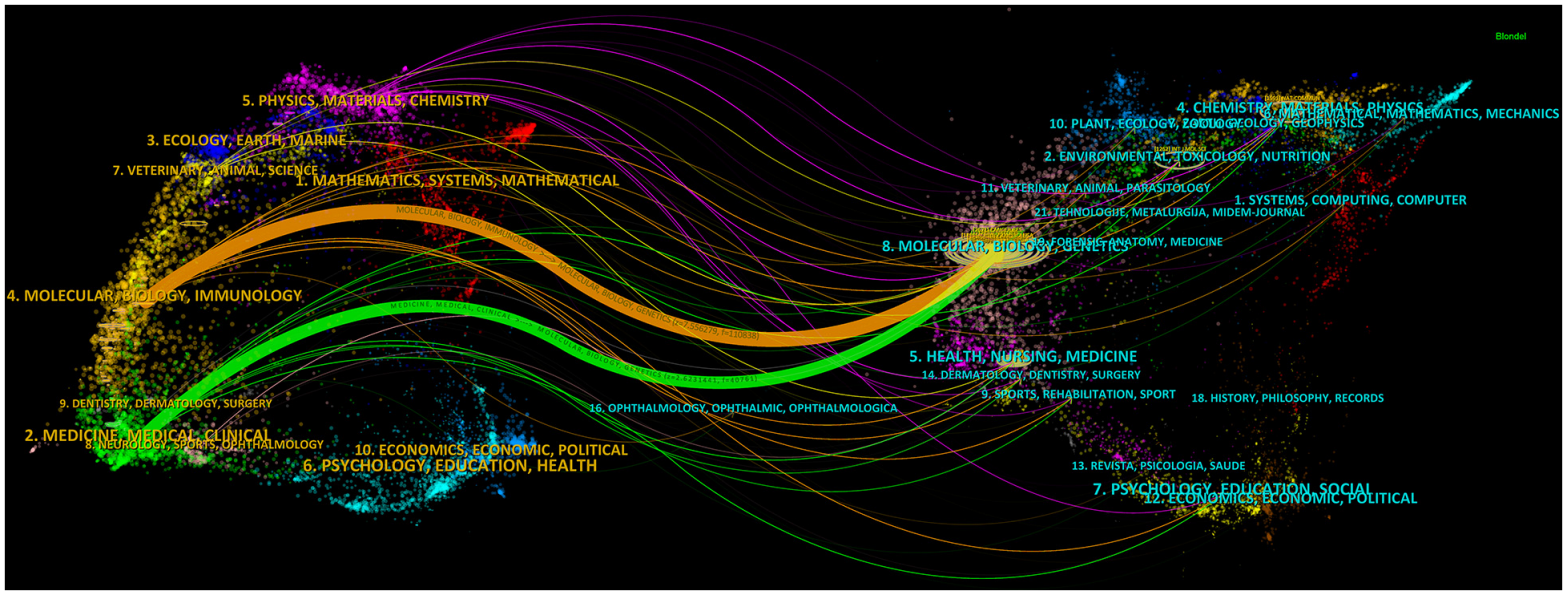
The dual-map overlay of journals on research of exosomes in lung cancer.

Out of the 521 journals where exosomes-related research on lung cancer was published, Cancers published the majority of papers (n=69), followed by International Journal of Molecular Sciences (n=54), Frontiers in Oncology (n=50), and Scientific Reports (n=35). For visualization purposes, we selected 20 journals with a minimum publication count of 14 (**Figure 7A**). **Figure 7A** illustrates that the International Journal of Molecular Sciences has positive citation relationships with Cancers, Oncogenes, and Frontiers in Oncology. As shown in **Table 3**, in the top 20 co-cited journals, four journals were cited more than 2,000 times, with Cancer Research (Co-citation = 2,671) being the most cited, followed by Plos One (Co-citation = 2,458), Oncotarget (Co-citation = 2,230), and Nature (Co-citation =2,173). We filtered the journals with a minimum co-citation of 800 and selected 25 journals for visualization (**Figure 7B**). **Figure 7B** demonstrates that Nature has co-citation relationships with Cell, Plos One, and Oncotarget. The double map overlay of journals reveals the citation relationships between journals and co-cited journals, with journal clusters on the left and cited journal clusters on the right. Labels in the map corresponded to the disciplines or fields related to the articles published in the journals. As shown in **Figure 8**, the orange pathway represents the main citation pathway, signifying that the articles published in molecular/biology/immunology journals in this study were mainly cited by molecular/biology/Genetics journals cited in the literature.

**Table 3:** Top 20 Journals and Co-cited Journals on research of exosomes in lung cancer.

Rank	Journal	documents	Co-cited Journal	Co-citation
1	Cancers	69	Cancer Research	2671
2	International Journal of Molecular Sciences	54	Plos One	2458
3	Frontiers in Oncology	50	Oncotarget	2230
4	Scientific Reports	35	Nature	2173
5	Oncotarget	33	The Proceedings of the National Academy of Sciences	1836
6	Cell Death & Disease	27	Molecular Cancer	1766
7	Cancer Letters	26	Scientific Reports	1755
8	Frontiers in Immunology	22	Cell	1720
9	Thoracic Cancer	22	Clinic Cancer Research	1716
10	Cells	20	Journal of Extracellular Vesicles	1663
11	Journal of Experimental & Clinical Cancer Research	20	Nature Communications	1593
12	Oncotargets and Therapy	20	Nature Cell Biology	1455
13	Frontiers in Cell And Developmental Biology	19	Oncogene	1336
14	Cancer Cell International	17	International Journal of Molecular Sciences	1264
15	Molecular Cancer	17	Cancer Letters	1237
16	Oncology Letters	16	Journal of Biological Chemistry	1211
17	Theranostics	16	Nature Reviews Cancer	1115
18	Biosensors & Bioelectronics	15	Cancer Cell	1087
19	Cancer Science	14	Science	1003
20	Translational Lung Cancer Research	14	International Journal of Cancer	999

### 3.6 Commonly cited references

**Figure 9:**
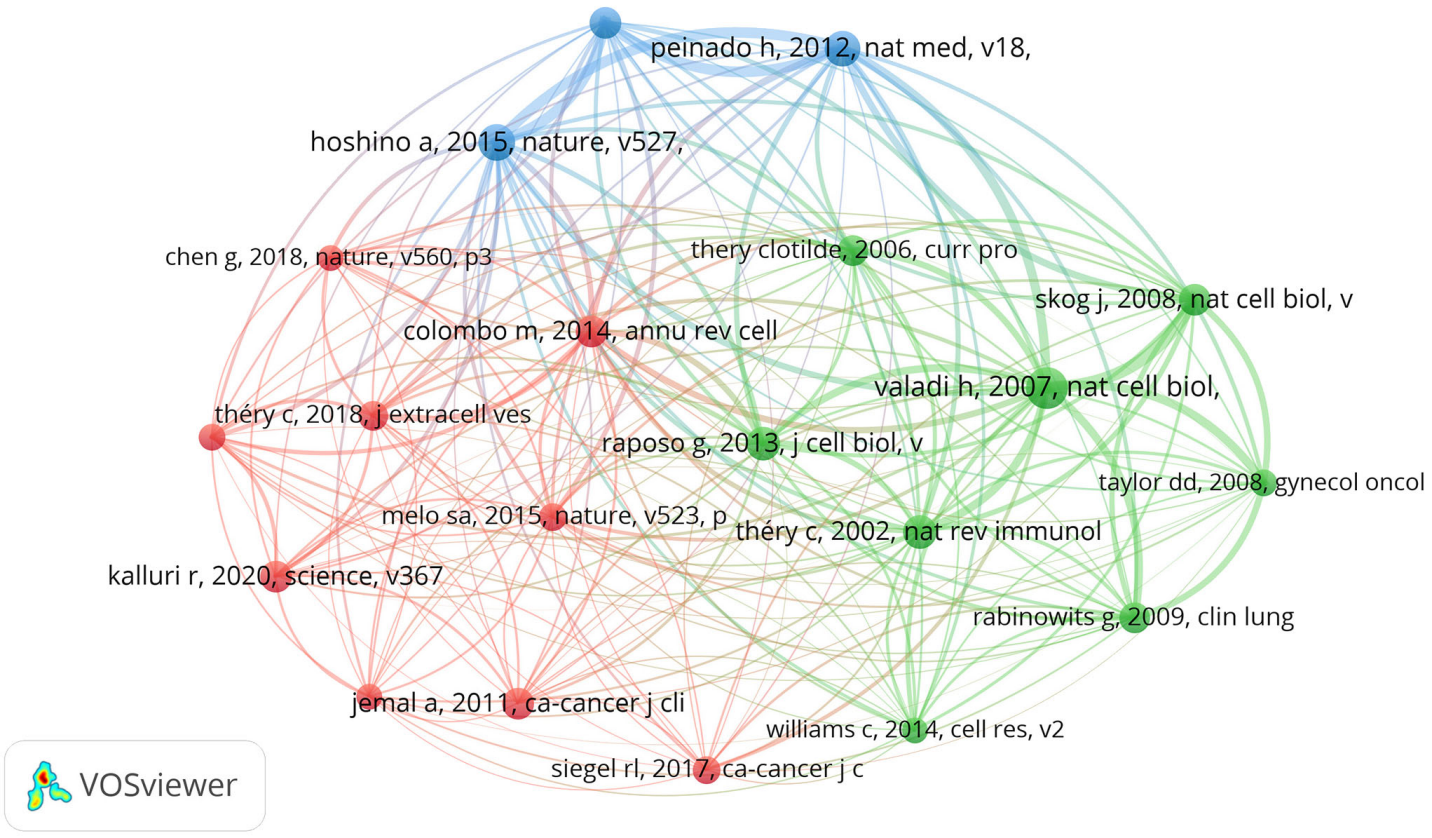
The visualization of co-cited references on research of exosomes in lung cancer.

In this study, we analyzed the citations of 1699 articles, totaling 71,961 citations. Of these, 20 articles had 100 or more citations. We visualized these 20 co-cited articles using VOSviewer (**Figure 9**), where each node represents a cited article. These 20 co-cited articles were classified into three clusters and distinguished by three colors. Additionally, we listed the top 10 most cited literature on exosomes in the field of lung cancer (**Table 4**), with the top three having more than 200 citations. From the literature co-citation network in **Figure 9**, it is evident that Hadi Valadi’s “Exosome-mediated transfer of mRNAs and miRNAs is a novel mechanism of genetic exchange between cells” is significantly co-cited with Graça Raposo’s “Extracellular vesicles: exosomes, microvesicles, and friends,” and Clotilde Théry’s “Exosomes: composition, biogenesis and function”.

**Table 4:** Top 10 co-cited references on research of exosomes in lung cancer.

Rank	Co-cited reference	citations
1	valadi h, 2007, nat cell biol, v9, p654, doi 10.1038/ncb1596	318
2	hoshino a, 2015, nature, v527, p329, doi 10.1038/nature15756	241
3	peinado h, 2012, nat med, v18, p883, doi 10.1038/nm.2753	216
4	théry c, 2002, nat rev immunol, v2, p569, doi 10.1038/nri855	188
5	raposo g, 2013, j cell biol, v200, p373, doi 10.1083/jcb.201211138	187
6	jemal a, 2011, ca-cancer j clin, v61, p134, doi [10.3322/caac.21492 10.3322/caac.20115 10.3322/caac.20107]	171
7	colombo m, 2014, annu rev cell dev bi, v30, p255, doi 10.1146/annurev-cellbio-101512-122326	167
8	kalluri r, 2020, science, v367, p640, doi 10.1126/science.aau6977	164
9	skog j, 2008, nat cell biol, v10, p1470, doi 10.1038/ncb1800	163
10	costa-silva b, 2015, nat cell biol, v17, p816, doi 10.1038/ncb3169	162

### 3.7 Analysis of research hotspots based on keyword co-occurrence

**Figure 10:**
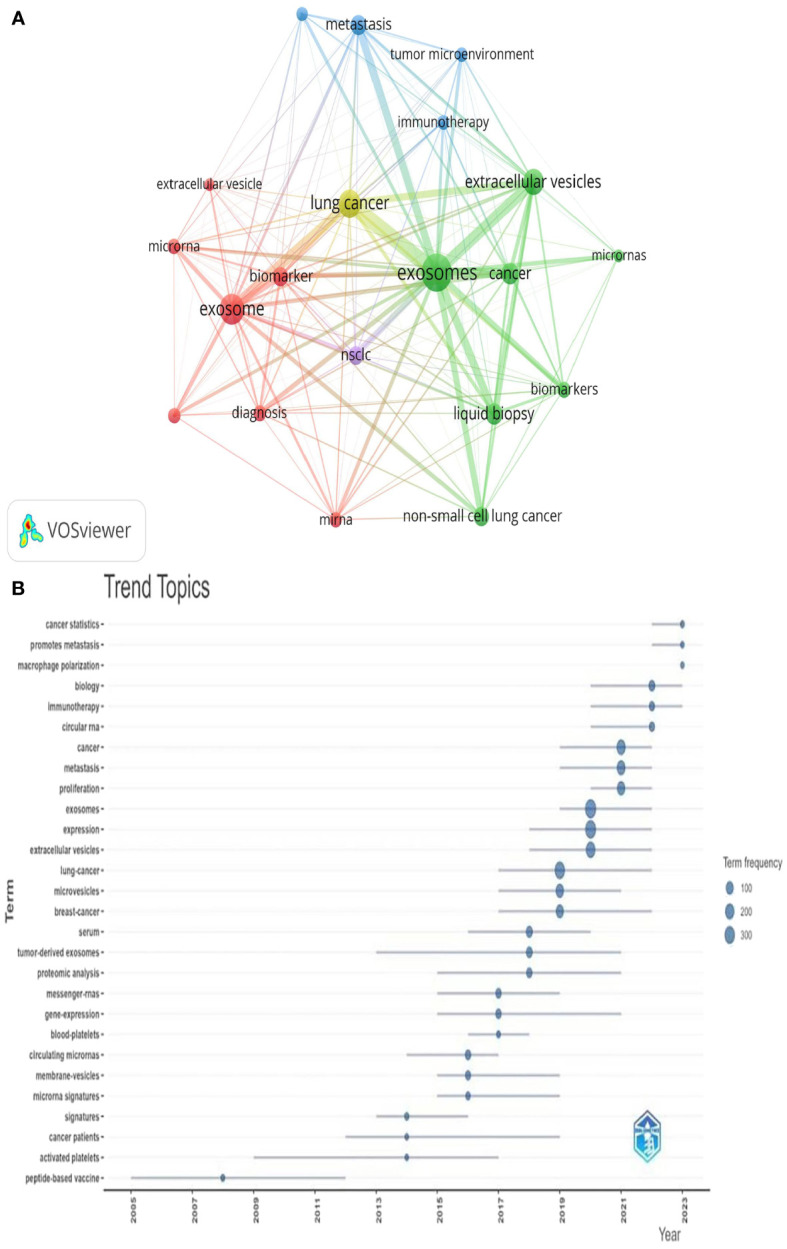
Keyword cluster analysis **(A)** and Trend topic analysis **(B)**.

**Figure 11:**
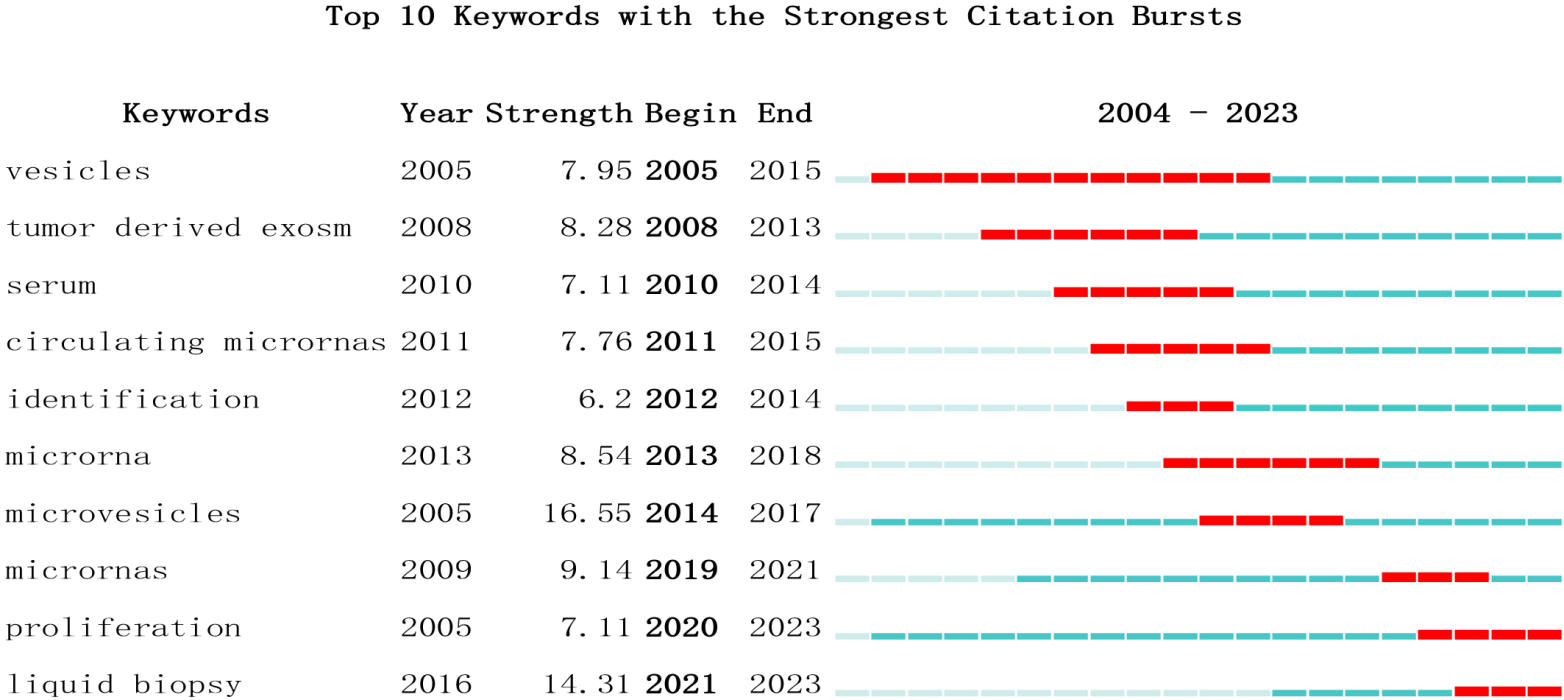
Top 10 Keywords with strong citation bursts. A red bar indicates high citations in that year.

Keywords serve as indicators of the interrelationships among various topics represented in the literature and encapsulate the essence of the article. Analyzing keywords is conducive to exploring the hotspots in the field. The keyword co-occurrence map of lung cancer-related exosomes shown in **Figure 10A** was generated using the VOSviewer software. The size of each node in this map corresponds to the frequency of the word, with the circle expanding as the frequency increases. Among keywords with a frequency of more than 34 occurrences, exosomes, extracellular, lung cancer and NSCLC exhibited the highest frequencies, with 827, 245, 242, and 190 occurrences, respectively (**Table 5**). To summarize the hot subjects of interest to scholars in the field of lung cancer exosomes, ten main directions emerge: cancer statistics, promotes metastasis, macrophage polarization, biology, immunotherapy, circular RNA, cancer, metastasis, proliferation, and exosomes (**Figure 10B**). The high-frequency keywords align closely with the top-ranked keywords in the center, indicating that higher frequencies correlate with more prominent centrality, covering hotspots and key turning points to a certain extent. Using CiteSpace, we identified the 10 most cited keywords. As shown in **Figure 11**, each red bar indicates a high citation rate in that year. The keyword with the strongest citation burst (strength=16.55) was microvesicles, with a citation burst as early as 2005 and a burst from 2014-2017. The second strongest citation burst (strength=14.31) occurred for the keyword liquid biopsy, bursting in 2021-2023. The keyword with the longest outbreak duration is vesicles, which lasted from 2005-2015. Generally, most keywords exhibit an outbreak intensity of 6.1-16.55 and an endurance intensity of 3 to 5 years. Keywords that persist from 2020 to the present include proliferation and liquid biopsy. These keywords reflect the development pulse and trends in lung cancer exosomes, with proliferation, liquid biopsy, and other keywords persisting until now, indicating that related research continues to be a key trend in the field of lung cancer exosomes.

**Table 5:** Top 15 keywords on research of exosomes in lung cancer.

Rank	keyword	occurrences	Rank	keyword	Centrality
1	exosomes	827	1	lung cancer	0.19
2	extracellular vesicles	245	2	exosm	0.16
3	lung cancer	242	3	proteomic analysis	0.11
4	non-small cell lung cancer	190	4	expression	0.1
5	biomarkers	164	5	cell derived exosm	0.08
6	cancer	131	6	breast cancer	0.07
7	liquid biopsy	129	7	extracellular vesides	0.06
8	metastasis	106	8	proteins	0.06
9	microRNA	95	9	tumor derived exosm	0.06
10	diagnosis	62	10	antitumor	0.06
11	lung adenocarcinoma	57	11	cells	0.05
12	miRNA	56	12	hepatocellular carcinoma	0.05
13	tumor microenvironment	52	13	down regulation	0.05
14	immunotherapy	50	14	dendritic cells	0.05
15	breast cancer	46	15	proliferation	0.04

### 3.8 Literature and citation explosion

**Figure 12:**
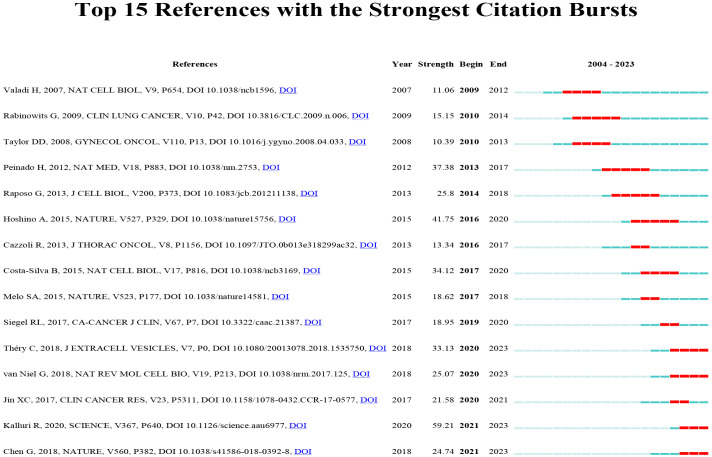
Top 15 references with strong citation bursts. A red bar indicates high citations in that year.

A citation burst refers to a phenomenon where specific references are frequently cited by researchers within a certain period. Using the CiteSpace tool, we identified 15 references that exhibited strong citation bursts (**Figure 12**). Each vertical bar represents a year, and the red bars indicate strong citation bursts. The citation burst phenomenon appears to date as far back as 2012. One of the references with the strongest citation burst (strength=59.21) is “The biology, function, and biomedical applications of exosomes,” published by Kalluri R. et al. in 2020. This study experienced a citation explosion during 2021-2023. The second strongest citation burst (strength = 41.75) was observed in the article titled “Tumor exosome integrins determine organotropic metastasis” published in Nature by Hoshino A. et al. This study continued to experience a citation explosion from 2016 to 2020. Overall, the intensity of these 15 references’ bursts ranged from 10.39 to 59.21, while the duration ranged from 2 to 5 years. Summarizing the main studies of these 15 references, **Table 6** provides detailed information based on the order of literature in **Figure 12**.

**Table 6:** The main research contents of the 15 references with strong citations bursts.

Rank	Strength	Main research content
1	11.06	Exosome-mediated transfer of mRNAs and microRNAs is a novel mechanism of genetic exchange between cells (8).
2	15.15	Exosomal MicroRNA may serve as a diagnostic marker for lung cancer (17).
3	10.39	MicroRNA signatures of tumor-derived exosomes as diagnostic biomarkers for ovarian cancer (18).
4	37.38	Exosome-mediated transfer of the oncoprotein MET as a key regulator of bone marrow mobilization, and metastatic progression (19).
5	25.8	Characterization of the extracellular vesicle and the mechanisms of its formation, targeting and function (20).
6	41.75	Exosomal integrins could be used to predict organ-specific metastasis (21).
7	13.34	MicroRNAs derived from circulating exosomes as non-invasive biomarkers for screening and diagnose lung cancer (22).
8	34.12	Exosomal migration inhibitory factor may be a prognostic marker for the development of pancreatic ductal adenocarcinomas liver metastasis (23).
9	18.62	Glypican-1 exosomes may serve as a potential non-invasive diagnostic and screening tool to detect early stages of pancreas cancer to facilitate possible curative surgical therapy (24).
10	18.95	Statistical analysis of cancer patients in the United States in 2017 (25).
11	33.13	Minimal information for studies of extracellular vesicles 2018 (26).
12	25.07	Shedding light on the cell biology of extracellular vesicles (27).
13	21.58	Evaluation of tumor-derived exosomal miRNA as potential diagnostic biomarkers for early stage non-small-cell lung cancer using next-generation Sequencing (28).
14	59.21	The biology, function, and biomedical applications of exosomes (29).
15	24.74	Exosomal PD-L1 contributes to immunosuppression and is associated with anti-PD-1 response (30).

## 4 Discussion

### 4.1 Growing trend

#### 4.1.1 Number of studies

Our examination of articles from the WOS revealed that the earliest research on lung cancer and exosomes was published in 2004, successfully extracting exosomes from pleural effusion of lung cancer patients for the first time. Research on lung cancer-related exosomes exhibited a gradual upward trend in the following 10 years, signifying considerable development in this field and laying a foundation for future explosive growth. Since 2015, there has been an exponential increase in literature related to lung cancer exosomes, indicating that the field has attracted much attention.

#### 4.1.2 International cooperation

Statistical visualization of the distribution of high-yield countries, institutions, and authors revealed that the countries leading in the publication of lung cancer exosome articles are China, the United States, and Italy; the top three high-yield research institutions, based on the number of articles published, are Nanjing Medical University, Shanghai Jiao Tong University, and the Chinese Academy Of Sciences. The top authors include Jing Li, Takahiro Ochiya, and Wei Wang. The collaboration among institutions and scholars appears to be relatively close. In the overall rankings of high-yield countries, institutions, and authors, China is ranked top, indicating its leadership in academic research on lung cancer exosomes. There is an increasing trend in international cooperation in lung cancer exosome research, with research teams collaborating across borders. This collaborative approach enhances communication and sharing of research results, thereby promoting the development of this field.

#### 4.1.3 Popular journals

An analysis of the journals in which exosome-related articles on lung cancer were published highlights Cancers as the predominant journal, underscoring its popularity in this field of research. Additionally, the International Journal of Molecular Sciences, Frontiers in Oncology, and Scientific Reports have over 30 publications. Among co-cited journals, prominent Q1 journals such as Cancer Research, Plos One, Oncotarget, and Nature stood out with over 2000 citations each. These journals signify high-quality international journals supporting research on lung cancer-related exosomes. Furthermore, current studies on lung cancer exosomes are mainly published in molecular, biology, and immunology-related journals. However, clinical journals are relatively few, indicating that the majority of studies are still in the basic research stage.

#### 4.1.4 Authors, co-cited authors and co-cited literature

In our analysis of core authors in the field of lung cancer exosomes, a total of 105 core authors were identified, with Jing Li, Takahiro Ochiya, and Wei Wang leading in paper publications, each contributing more than 10 papers. Jing Li et al.’s primary focus lies in the roles of exosome-associated mRNAs in lung cancer. Their research highlighted that overexpression of miR-193a-3p inhibited lung cancer cell proliferation and invasion, promoting apoptosis, whereas reduction of epidermal growth factor receptor-4 (ErbB4) mimicked the induction of miR-193a-3p. miR-193a-3p treatment demonstrated *ex vivo* anti-tumor effects by negatively regulating ErbB4. Consequently, ErbB4 emerges as a potential novel target for lung cancer therapy. The significance of miR- 193a-3p and ErbB4 as important regulators within cellular pathways suggests their potential as attractive and novel therapeutic targets for lung cancer treatment (31). Subsequently, the authors analyzed the molecular characteristics of Lewis lung carcinoma (LLC) exosomal proteins and mRNAs, establishing the foundation for exploring diagnostic markers for lung cancer. Moreover, the authors analyzed the interactions between exosome membrane proteins and their target proteins, unveiling the potential tissue predisposition of LLC cell-derived exosomes. These findings provide a theoretical basis for studying exosome-mediated tissue targeting and distal lung cancer metastasis (32). In another study focusing on lung cancer exosomes, elevated levels of circulating miR-520c-3p and miR-1274b were identified in NSCLC patients. Following tumor resection, there was a significant decrease in the levels of these two miRNAs. These observations highlight the potential use of miR-520c-3p and miR-1274b as biomarkers for NSCLC (33). Takahiro Ochiya et al. provide an in-depth summary of the impact of circulating miRNAs encapsulated in EVs on lung cancer research. The role of exosomal miRNAs in cell-cell communication is thoroughly discussed, and the validity of these elements as predictive biomarkers for cancer malignancy is explored in-depth (34, 35). Wei Wang’s research delves into NSCLC exosomes and their role in promoting proliferation, phagocytosis, and secretion of microglia in the metastatic microenvironment via exosomal miRNAs (36), and mesenchymal stem cell-mediated tumor-targeted drug delivery therapies (37). His study revealed that human bone marrow mesenchymal stem cell (MSC)-derived exosomes, containing miRNA-425, play a role in promoting migration, invasion, and lung metastasis by down-regulating cytoplasmic polyadenylation binding protein 1 (38). Furthermore, the sorting of miR-122-5p by extracellular vesicles (EVs), mediated by heterogeneous nuclear ribonucleoprotein A2B1, is implicated in promoting lung cancer progression (39). Additionally, exosomal miRNA-223-3p derived from tumor-associated macrophages promotes lung metastasis in breast cancer 4T1 cells (40). Exosomal miRNA-26b-5p is found to downregulate activating transcription factor 2, enhancing radiosensitivity in lung adenocarcinoma cells (41). The study also highlights the role of Circ_0002476 in regulating cell growth, invasion, and mitochondrial DNA damage in NSCLC by targeting the miR-1182/mitochondrial transcription factor A axis (42).

In the analysis of co-cited authors and documents, four emerged with more than 100 co-citations, and the most frequently co-cited author is Clotilde Théry with 502 citations. Théry’s primary research focus revolves around the immunological mechanisms and role of exosomes in lung cancer, displaying a keen interest in the interplay between exosomes and tumor growth. In 2015, Théry published a seminal work titled “Dendritic cell-derived exosomes as maintenance immunotherapy after first-line chemotherapy in NSCLC,” demonstrating the capacity of dendritic cell-derived exosomes to enhance anti-tumor immunity in NK cells among patients with advanced NSCLC (43). The following year, Théry published a review in the journal Cell titled Communication by Extracellular Vesicles: Where We Are and Where We Need to Go.” This comprehensive review delves into the field of tumor cells and their microenvironment, with results and challenges applicable to both physiological and pathological systems where extracellular vesicle-regulated communication occurs (44). The co-citations analysis included 1699 within the study, revealing 20 articles with over 100 citations, and the top 3 articles having more than 200 citations each, indicating that these articles have high impact value in lung cancer-related exosomes. Hadi Valadi’s 2007 Nature Cell Biology article “Exosome-mediated transfer of mRNAs and miRNAs is a novel mechanism of genetic exchange between cells” is the most cited article. This article elucidates the concept that exosome-mediated transfer of mRNAs and miRNAs is a novel mechanism of genetic exchange between cells, laying the foundation for subsequent investigations into the effect of exosomal mRNAs and miRNAs on tumors. Simultaneously, it provides a crucial method for extracting exosomal mRNAs and miRNAs, significantly contributing to the field of exosome-related research. The role of exosome-related research is enormous (8).

#### 4.1.5 Interdisciplinarity

Multidisciplinary intersection plays an important role in lung cancer exosome research. Lung cancer exosomes, serving as an intercellular communication medium, significantly contribute to the occurrence and development of lung cancer. The use of a multidisciplinary research approach proves instrumental in understanding biological functions, biomarkers, and immunoregulatory mechanisms associated with lung cancer exosomes. This approach opens up avenues for advancements in early diagnosis, personalized treatment, and immunotherapy for lung cancer. The study of lung cancer exosomes draws upon knowledge and techniques from diverse disciplines, including oncology, cell biology, molecular biology, and bioinformatics. Researchers within the field of oncology leverage the biological functions of lung cancer exosomes to investigate tumor metastasis and drug resistance mechanisms. For instance, You J et al. found that tumor exosomes influence lung cancer metastasis by delivering snail family transcriptional repressor 1, thereby promoting cellular epithelial-mesenchymal transition (45), Similarly, Lobb RJ et al. found that lung cancer mesenchymal cell exosomes, containing Zinc finger E-box binding homeobox 1 (ZEB1) mRNA, increases ZEB1 expression in recipient cells, inducing their transformation from an epithelial to mesenchymal phenotype and promoting bronchial epithelial cell metastasis resistant to chemotherapy (46). In the field of cell biology, researchers investigate the mechanisms underlying exosome generation and release in lung cancer. Notably, Qing-Fang H et al. published a review entitled “Exosome biogenesis: machinery, regulation, and therapeutic implications in cancer,” offering a comprehensive overview of exosome generation mechanisms and their regulation in tumors. The review underscores the potential of targeting exosome generation as a promising strategy for tumor therapy (47). Molecular biology researchers focus on analyzing biomarkers and signaling pathways within lung cancer exosomes. An example is Lin Shi et al. who identified that cancer-associated fibroblast-derived exosome miRNA-20a inhibits the phosphatase and tensin homolog of chromosome 10 (PTEN)/phosphatidylinositol 3-kinase (PI3K)/protein kinase B (PKB, Akt) pathway, consequently promoting the progression of NSCLC and chemoresistance (48). Bioinformatics researchers contribute to lung cancer exosome research by using big data analytics to mine potential information. For instance, Jin XC et al. used next-generation sequencing to evaluate tumor exosomal miRNAs as potential diagnostic biomarkers for early-stage NSCLC (28). This multidisciplinary research approach facilitates a comprehensive understanding of the mechanisms through which lung cancer exosomes contribute to the development of lung cancer. It not only generates diverse insights but also offers numerous possibilities for the diagnosis and treatment of lung cancer. Consequently, future research on exosome in lung cancer will increasingly multidisciplinary collaboration, and promote communication and cooperation among different disciplines to advance the exosome research field in lung cancer.

### 4.2 Research hotspot

Keyword analysis can reveal the changes in the developmental trends of a specific research field and reveal the research hotspots in a specific field. This analytical method can identify the key features and research direction in the field by assessing keyword co-occurrence, keyword clustering, keyword occurrence frequency, and theme word analysis. In the following section, the research hotspots based on keyword co-occurrence will be discussed.

#### 4.2.1 Biological functions of exosomes in lung cancer

In recent years, several studies have explored the biological functions of lung cancer exosomes. Exosomes from lung cancer cells serve as an important information transfer medium and have been shown to play an important role in lung cancer metastasis and drug resistance. For example, tumor exosomes can regulate lung cancer metastasis by promoting the transformation of cellular epithelial to mesenchymal transition (EMT). Kim E et al. found that Tumor necrosis factor receptor-associated factor 4, which is also expressed in lung fibroblasts surrounding NSCLC cells, can promote the proliferation and EMT of NSCLC cells by stabilizing the NADPH oxidase complex (49). Xiaoyin Z et al. reported that the human umbilical cord MSCs enhanced EMT, invasion, and migration, while suppressing the proliferation and stimulating apoptosis of lung cancer cells. Knockdown of the transforming growth factor-beta1 expression in human umbilical cord MSCs reversed the exosome-mediated activated EMT in lung cancer cells (50). Furthermore, tumor-derived exosomes can regulate the metastasis of lung cancer by affecting cell proliferation, apoptosis, and migration. A study by Hui X et al. demonstrated that exosomes derived from particulate matter 2.5-associated lung cancer cells could enhance the proliferation of lung cancer cells through the Wnt3a/β-catenin pathway (51). Nan Z et al. found that circSATB2 was highly expressed in NSCLC cells and tissues. It was reported that circSATB2 positively regulated the expression of fascin homolog 1, actin-bundling protein 1 (FSCN1) in lung cancer cells via miR-326. In addition, circSATB2 can stimulate the proliferation, migration, and invasion of NSCLC cells and induce abnormal proliferation of normal human bronchial epithelial cells via the exosomal transfer (52). Several factors in lung cancer exosomes can affect the TME, regulate inflammatory responses, angiogenesis, and immune escape, thus promoting tumor development. A study by Kim D et al. demonstrated that exosomes released by lung cancer cells contain high levels of programmed cell death ligand 1 (PD-L1). This decreases T-cell activity, induces immune escape, leading to enhanced tumor growth. PD-L1 in exosomes inhibited the secretion of interferon-gamma by T cells. In a previous study, exosomes suppressed cytokine production, induced apoptosis of CD8+ T cells, impaired immune function, and promoted lung cancer metastasis (53). A study by Hsu Y et al. showed that miR-23a was upregulated in lung cancer exosomes under hypoxic conditions. This inhibits prolyl hydroxylases 1 and 2, leading to the accumulation of the hypoxia-inducible factor-1α in endothelial cells. The release of miR-23a from hypoxic lung cancer cells favors angiogenesis and cancer cell metastasis. In the study, inhibition of miR-23a suppressed angiogenesis and tumor growth in a mouse model. Results showed that in lung cancer patients, increased circulating miR-23a levels were positively correlated with pro-angiogenic activity (54).

#### 4.2.2 Biomarkers in lung cancer exosomes

Since 2004, researchers have explored biomarkers in lung cancer exosomes following the pioneer publication by Martin P et al. In their study, sucrose gradient ultracentrifugation was employed to isolate exosomes from malignant pleural effusions and determine their proteomic content (55). In the last decade, investigations into biomarkers in lung cancer exosomes have intensified. The key research hotspots include lung cancer diagnosis, prognosis assessment, targeted therapy, and disease monitoring.

Biomarkers in EVs can facilitate early diagnosis of lung cancer and that of other diseases. A study conducted by Rabinowits et al. found that EV miRNAs in NSCLC patients are highly similar to those found in NSCLC tissues, suggesting that EV miRNAs can serve as markers for liquid biopsy in NSCLC (17). A clinical study performed by Marc R. and colleagues found that YKT6 can regulate the release of EVs from lung cancer cells and is precisely controlled by miR-134 and miR-135b (56). Moreover, Kamada H and their research group found that the epidermal growth factor receptor expressed on the EV membrane may be a potential biomarker for lung cancer diagnosis (57). Another clinical study by Dong H et al. demonstrated that EV miR-619-5p promotes the growth and metastasis of NSCLCs by inhibiting RCAN1.4 and can serve as a diagnostic marker for these lung cancers (58). EV-associated biomarkers have the potential to help assess the prognosis of lung cancer patients, including disease progression and prediction of treatment response (59, 60). Hitoshi D et al. found that the concentration level of plasma EV miR-21 and mir-4257 can be used to predict the recurrence of NSCLC following curative resection (61). In the study by Marc R, results showed that the level of YKT6 can influence the prognostic outcomes of NSCLC patients following surgery, higher levels were predicted to have shorter disease-free survival and overall survival (56). Biomarkers in exosomes that have prognostic value in lung cancer include let-7a-5p, miR-21, miR- 106b, miR-486-5p, miR-23b-3p, miR-10b-5p, and miR-21-5p (62–66).

Such biomarkers may serve as a guidance for the development of therapeutic strategies. They can also be used to evaluate the sensitivity and resistance to specific treatment approaches. Detecting exosomes in the TME and observing changes in biomarkers of lung cancer patients during treatment can help assess disease progression and treatment efficacy. A study by LK W et al. identified melanoma differentiation-associated gene-9 (MDA-9)/Syntenin as a novel therapeutic target that can influence cancer progression and exosome biogenesis. Lung adenocarcinoma patients with high MDA-9/Syntenin and high Slug expression tend to have poor overall survival compared with those with low expression. Studies have shown that MDA-9/Syntenin is a key articulator of Slug, which transcriptionally promotes Slug-mediated EMT thereby accelerating cancer invasion and metastasis (67).

#### 4.2.3 Liquid biopsy and immunotherapy

Liquid biopsy, as a non-invasive method, can be obtained from diverse samples, unlike traditional tissue biopsy. Moreover, it is easier to obtain and is not affected by spatial and temporal heterogeneity of tumors making it ideal for real-time monitoring of tumors. It is, therefore, applied in early screening and diagnosis of lung cancer, precision treatment, efficacy monitoring, analysis of drug resistance, and prognosis assessment. Numerous studies have shown that some components of lung cancer exosomes can be used as markers in liquid biopsy, such as miRNAs (mit-379, mir-378a, mir-139-5, and mir-200-5p) in exosomes can be used for differential diagnosis of lung cancer patients (68). Moreover, miRNAs in exosomes can be used as biomarkers to distinguish NSCLC types. For instance, exosomes carrying miR-30e-3p, miR-30a-3p, miR-181-5p, and miR-361-5p are specific diagnostic markers for lung adenocarcinoma. On the other hand, exosomes containing miR-15b-5p, miR-10b-5p, and miR-320b can be utilized as specific diagnostic markers for squamous lung cancer (28). Extraction and purification techniques play a crucial role in exosome liquid biopsy. Although some methods have been developed for exosome isolation based on density size, surface molecular markers, charge affinity, and microfluidic chips (69), the time-consuming and laborious steps of isolation and purification, which are difficult to quality-control and standardize, are still a major limiting factor in the clinical application of exosome liquid biopsy (70).

In recent years, immunotherapy has been one of the major advances in the treatment of advanced tumors, with NSCLC patients benefiting from this approach. In addition, biomarkers such as PD-L1, tumor-infiltrating lymphocytes, tumor mutational load, mutations in certain driver genes, and microsatellite instability/defective mismatch repair are considered to be accurate predictors of the efficacy and prognosis of immunotherapy (71). Exosomal PD-L1 can dynamically display the average expression level of PD-L1 and may serve as a potential biomarker for immunotherapy (30). Yang et al. (72) found that NSCLC patients with changes in exosomal PD-L1 at 2 months of treatment with ICIs that were higher than at baseline had better progression-free and overall survival. Nevertheless, Del et al. (73) demonstrated a significant down-regulation of plasma-derived exosomal PD-L1 in NSCLC patients responding to treatment, while an elevation was observed in individuals experiencing disease progression, a finding corroborated by another study (74). In addition, Shimada et al. (75) found that baseline exosomal PD-L1 could distinguish responders from non-responders. In the future, the predictive value of exosomal PD-L1 needs to be further investigated. In addition, three miRNAs of the hsa-miR-320 family were identified as potential biomarkers for selecting advanced NSCLC patients to receive immunotherapy, with the exosomal miRNA miR-320d considered to be the most significantly differentially expressed miRNA between patients with progressive disease and those in partial remission (76). In addition, exosome-based liquid biopsies are less invasive and less costly than tissue biopsies (77). With the development of liquid biopsy technology, exosomes have the potential to become a potential biomarker for immunotherapy.

### 4.3 Progress and prospects of lung cancer exosomes research

#### 4.3.1 Progress in research

In recent years, significant progress has been made in the study of lung cancer exosomes, with broad implications for diagnosis, treatment, and understanding of the underlying mechanisms of the disease. One of the most promising areas of exosome research in lung cancer is biomarker discovery. Exosomes contain a variety of molecular components of their cell of origin, including DNA, RNA (miRNA, mRNA, and non-coding RNA), proteins, and lipids, which can be used for the early detection of lung cancer, providing a potential non-invasive diagnostic tool. Also, these molecular markers change along with the development of lung cancer and their levels in body fluids reflect the presence and extent of tumor progression. He Z et al. found that exosomal long-chain non-coding RNA TRPM2-AS promotes cancer angiogenesis through the NOTCH1 signaling pathway, suggesting its potential as a diagnostic biomarker to improve early diagnosis and survival outcomes in lung cancer (78). Xu W et al. found that exosome circ_0000735 was upregulated in NSCLC and its knockdown inhibited the proliferation and metastasis of NSCLC cells, suggesting its role in NSCLC diagnosis and as a therapeutic target (79). Alper B et al. used a novel sensor platform with magnetic levitation to capture exosomal membrane proteins (ExoMPs) EpCAM, CD81, and CD151 using antibody-functionalized microspheres as markers for cancer exosomes, exosomes, and NSCLC-derived exosomes, respectively, which shows promise in detecting and differentiating lung cancer-associated exosomes and offers new pathways for biomarker-based diagnosis, providing a new avenue for biomarker-based diagnosis (80). Hayato K et al. found that preoperative markers derived from extracellular vesicles were associated with lung cancer recurrence, suggesting their utility in prognosis (81). Tao X et al. Comprehensive analysis of the exosome gene LYPD3 pointed out its role in lung cancer prognosis and immune cell infiltration, signaling its importance in early screening and detection (82). Bingbing Y et al. conducted a systematic review of the diagnostic and prognostic value of exosomal microRNAs in lung cancer and found that exosomes miR-486-5p and miR-451a could be used as new diagnostic biomarkers for lung cancer (83). Exosome studies have revealed the molecular mechanisms underlying lung cancer progression and metastasis. Exosomes promote tumor growth by regulating the tumor microenvironment, promoting angiogenesis, and inhibiting immune responses to tumors. For example, exosome circ_0000735 is involved in the proliferation and metastasis of NSCLC cells (79). Ren Z et al. found that hypoxia-generated exosomes promote lung adenocarcinogenesis by regulating HS3ST1-GPC4-mediated glycolysis (84). Xiu R H et al. found that exosomal long non-coding RNA MLETA1 promotes tumor progression and metastasis in NSCLC by regulating miR-186-5p/EGFR and miR-497-5p/IGF1R axis (85). In the therapeutic field, exosomes also show great potential. Due to their ability to efficiently transport and deliver a variety of bioactive molecules *in vivo*, exosomes are being investigated as carriers for carrying drugs, gene editing tools (e.g. CRISPR-Cas9), or other therapeutic molecules directly to tumor cells. The advantage of this approach is its high targeting and low immunogenicity, providing a new strategy for lung cancer treatment. Rae K et al. conducted a novel monoclonal antibody targeting TM4SF4 enhanced anti-tumor activity in NSCLC by modulating the immune checkpoint ligand and exosomal pathways (86).

#### 4.3.2 Perspectives on exosomes in lung cancer

EVs are gaining prominence in the field of lung cancer research due to their potential for early detection, diagnosis, and targeted treatment of the disease. These nano-sized vesicles are secreted by a variety of cell types and have the unique ability to encapsulate a variety of biomolecules, cross biological barriers, and are designed to target specific molecules. This makes them very promising for the development of diagnostic markers and precise drug delivery systems for cancer cells (87). Lung cancer, especially at advanced stages, often poses significant challenges for effective treatment. Most lung cancer patients are diagnosed at an advanced stage, complicating surgical resection and increasing the risk of postoperative recurrence. The continuous development of lung cancer treatment has led to an increasing interest in precision therapy, especially in-depth studies of exosomes in lung cancer, which have made more immunotherapeutic and targeted therapeutic approaches possible (5).

The role of exosomes in lung cancer extends beyond their biological function to their potential application in the clinical setting. Their stability, biocompatibility, and ability to penetrate biological barriers, while exhibiting low toxicity and immunogenicity, make them a valuable tool in cancer therapy. Exosomes can influence tumor progression, angiogenesis, cell growth and migration, and are being explored as diagnostic and prognostic biomarkers, contributing to the development of minimally invasive diagnostic methods and next-generation therapies (5). In the context of NSCLC and EGFR mutant lung cancer, exosomes show promise in liquid biopsy applications. Exosomes can be efficiently detected in a variety of body fluids and contain genetic information associated with tumors, providing a non-invasive method to gain genetic insights into disease. Their use in liquid biopsy can facilitate early detection of tumors, contribute to early diagnosis, and assist in clinical treatment, potentially improving the prognosis and quality of life of lung cancer patients (88). In addition, exosomal protein and nucleic acid markers are important for early diagnosis and prognosis of lung cancer. Exosomes carry specific proteins and miRNAs that are indicative of the physiological and pathological states of cancer cells. Identifying these markers can lead to the development of targeted therapies and innovative diagnostic strategies, marking a step forward in personalized medicine for lung cancer.

In summary, significant progress has been made in exosome research in lung cancer, providing new insights into the disease and paving the way for new diagnostic and therapeutic approaches. With ongoing research able to address current challenges, exosome-based lung cancer strategies are promising and may change the landscape of lung cancer diagnosis and treatment. In the future, more attention should be paid to multidisciplinary intersections and international collaborations, biological functions of lung cancer exosomes, biomarker identification, and immunomodulatory mechanisms to provide more strategies for early diagnosis, individualized treatment, and immunotherapy of lung cancer. Researchers should aim to solve the current problems in this field. For example, how tumor cells utilize the biological information in exosomes and how exosomes affect their surrounding microenvironment still need to be thoroughly investigated. Therefore, more basic experimental and clinical studies on exosomes in lung cancer are still needed to better understand their biological properties and clinical application prospects, and to provide more effective strategies and tools for the effective treatment and management of lung cancer.

## 5 Advantages and limitations

In this bibliometric analysis of lung cancer exosomes, we demonstrate the current research status as well as the research hotspots in the field. Our results provide an important reference for future studies. In addition, the studies included in this paper are visualized, which intuitively presents the information and data, thereby making it easy for readers to understand the content of the article more quickly and comprehensively. Thirdly, all our studies are based on conclusions drawn from data analysis, therefore, our results are objective. While our study provides valuable insights, it is not without limitations. Our screening process may have overlooked relevant non-English articles, and our evaluation of high-level studies may have underestimated their academic value due to the challenges of generalization. We only included the Web Science Core database in the article search and should have included more search engines such as PubMed, SCOPUS and Google Scholar, which cover a wider range of subject areas and can provide a more comprehensive view of research results. In addition, search engines such as PubMed and Google Scholar are able to show the number of citations to research literature, which helps to assess the impact and importance of the research.

## 6 Conclusion

As an emerging research field, lung cancer exosomes have a broad research prospect. Publications on lung cancer exosomes have shown a rising trend year by year, and research on lung cancer exosomes has received more and more attention from scholars all over the world. China and the United States rank first and second in the number of publications. However, there is insufficient academic learning cooperation and exchange between the two sides, and Chinese universities account for a large proportion of research institutes in this field. Jing Li is the most productive author, Clotilde Théry is the most co-cited author, and Cancers is the journal with the highest number of publications. The current focus in the field of lung cancer exosomes is on biomarkers, liquid biopsies, immunotherapy, and tumor microenvironment.

## Data availability statement

The original contributions presented in the study are included in the article/supplementary material. Further inquiries can be directed to the corresponding authors.

## Author contributions

WZ: Investigation, Methodology, Writing – original draft, Writing – review & editing. XFZ: Investigation, Methodology, Writing – review & editing. XBZ: Visualization, Writing – review & editing. YX: Investigation, Writing – review & editing. ML: Methodology, Writing – review & editing. XY: Resources, Writing – review & editing. YJ: Visualization, Writing – review & editing. XS: Funding acquisition, Writing – review & editing.
